# The production and exportation of artemisinin-derived drugs in China: current status and existing challenges

**DOI:** 10.1186/s12936-016-1422-3

**Published:** 2016-07-15

**Authors:** Yangmu Huang, Hui Li, Danlu Peng, Yu Wang, Qiaomeng Ren, Yan Guo

**Affiliations:** School of Public Health, Peking University, Xueyuan Road 38, Haidian District, Beijing, 100191 China; China Chamber of Commerce for Import & Export of Medicines & Health Products, Floor11-12, Building 3, Beijing INN, No.6 Nanzhugan Hutong, Dongcheng District, Beijing, 100191 China

**Keywords:** Artemisinin, Active pharmaceutical ingredients, Finished pharmaceutical products, Production volume, Export value, Prequalification certification, China

## Abstract

**Background:**

As the discoverer and a major manufacturer of artemisinin, China has made valuable contributions to malaria control and the global market of artemisinin-derived drugs. This study aims to explore the production and export status of artemisinin-derived drugs in China during 2011 and 2014 to provide a clear understanding of China’s participation in this field and also to provide guidance for its future role on global malaria control.

**Methods:**

Production and exportation data were obtained from the Ministry of Industry and Information Technology (MIIT) database of the People’s Republic China and monthly reports of the General Administration of Customs of China, respectively. The production volume, export volume, export value, and export area of artemisinin and its derivatives (artemether, artesunate, dihydroartemisinin), including both active pharmaceutical ingredients (APIs) and finished pharmaceutical products (FPPs), were descripted and analysed by Microsoft Excel.

**Results:**

Between 2011 and 2013, the total production volume of artemisinin-derived APIs and FPPs reached 543.41 metric tons (MT) and 14.79 MT, respectively. The total export value and volume of artemisinin-derived APIs during 2012 and 2014 was US$ 211.39 million and 338.53 MT; the total export value and volume of FPPs was US$ 227.17 million and 4401.44 MT. Compared with the sharply decreasing trend of API exports each year, the export value of FPPs kept at a more stable level, with 14.66 % increase in 2013 and 5.31 % decrease in 2014. As for exportation areas, India was the largest purchaser of APIs, accounting for nearly a half of the total amount, while FPPs mainly exported to African countries.

**Conclusions:**

Exports of artemisinin derivatives for China have been transforming from API-dominated to FPP-dominated. However, the exportation of artemisinin-derived drugs in China still face the challenges of small market share in the global FPP market and indirect sale of FPPs through a third country due to the deficiency in obtaining relevant certification into global market.

## Background

The year 2015 witnessed the phased success of malaria control in halting and reversing malaria incidence for Millennium Development Goal 6C. However, the disease burden is still heavy with an estimated 214 million cases and approximately 438,000 deaths occurring in 2015 and almost half of the world’s population living at risk, especially in sub-Saharan Africa [1]. Termed as one of the three major public health problems worldwide, malaria is the top cause of morbidity from neglected diseases and accounts alone for 82.7 million disability adjusted life year (DALYs) in the developing world in 2010 [2, 3]. Despite vaccine candidate RTS,S (also known as Mosquirix™), no vaccine is available for prevention of any human malaria infection. In the immediate future, malaria control by medical products will still mostly depend on treatment with adequate and effective anti-malarial drugs [4]. Given the emerging issue of artemisinin resistance, research and development of non-artemisinin-based anti-malarial medicines that may take over from artemisinin derivatives in the medium term are in need. Luckily, there is a pipeline of more than 10 new anti-malarial drugs in various stages, including ones with non-artemisinin compounds.

Among available anti-malarial drugs, artemisinin and its derivatives (mainly artesunate, artemether, artemotil, dihydroartemisinin) have the superiority of rapid clearance of parasitaemia and can delay the spread of drug resistance when used in combination with another effective partner drug. In 2001, artemisinin-based combination therapy (ACT) was recommended by World Health Organization (WHO) as the best current treatment of uncomplicated falciparum malaria [5]. Since 2013, 79 countries with malaria epidemic designate ACT as first-line anti-malarial drugs [1]. Research showed that between 2000 and 2015 at least 145 million clinical cases were averted by adopting ACT across Africa. [6]. The promotion of ACT boosts demand for artemisinin-derived active pharmaceutical ingredients (APIs) and finished products.

China has played an essential role in the discovery and manufacturing of artemisinin. The 2015 Nobel Prize in Physiology was awarded to a Chinese scientist, Tu Youyou, for the discovery of artemisinin (qinghaosu) as a tribute and acknowledgement to China’s contribution in this field. Since China started the Project 523 for anti-malarial medications in 1967, efforts has been made in the research and development of artemisinin for almost half a century [7]. Moreover, China is a major manufacturer of artemisinin and its derivatives, with an integrated industry covering *Artemisia annua* planting, exaction, research and development, drug production, and sale [8]. In the artemisinin manufacturing industry, Chinese companies mainly provide APIs for foreign pharmaceutical companies to produce final dosages and accounts for 85 % of the world market, while the market share of finished pharmaceutical products (FPPs) is quite small and only one company (Guilin Pharmaceutical Shanghai Corporation) is prequalified for providing artemisinin-derived dosages [9].

In the past 5 years, no published article was found on the production and export status of artemisinin and its derivatives in China using authoritative national data. Understanding the current status and existing gaps in anti-malarial products is necessary. This study used national data from China’s Ministry of Industry and Information Technology (MIIT) database and reports of the General Administration of Customs (GAC) to analyse the production and export of artemisinin and its derivatives (artemether, artesunate, dihydroartemisinin) between 2012 and 2014, including APIs and FPPs. The main purpose of this study is to provide potential guidance for future development of artemisinin-derived drugs to ensure China’s better participation in global malaria control and elimination.

## Methods

### Data source and data description

The production data of APIs and FPPs of artemisinin and its derivatives (artemether, artesunate, dihydroartemisinin) in China between 2011 and 2013 were obtained from Chinese government statistics reported via MIIT. MIIT requires certain manufacturing enterprises to self-report their production data each year. This is the national official database of domestic production in China and the data used by Chinese policymakers. However, due to the limitation of self-report, the production data of dihydroartemisinin piperaquine compound tablets and artemether capsule were only reported in 2011.

With the assistance of China Chamber of Commerce for Import and Export of Medicines and Health Products, export data were derived from China GAC. Relevant data included export volume, export value and export areas (countries and continents) of APIs and FPPs of artemisinin and its derivatives between 2012 and 2014. The export volume of dihydroartemisinin API was quite small and not included in this study. Since the export volume (weight) and value of each batch are recorded in detail by GAC during exportation, export data derived from GAC are more objective in reflecting the total production and export situation of artemisinin and its derivatives. Thus, this article will mainly focus on the analysis of export data of artemisinin-derived APIs and FPPs.

### Data analysis

All the data were double-entered into Microsoft Excel and checked for consistency. Microsoft Excel was used for analysis and description. Microsoft Excel was also used for data analysis and description. The production volume and export volume were reported in metric tons (=1000 kg); the export value was reported in US$ or tens of millions, calculated according to the exchange rate in that year.

## Results

### Production of artemisinin-derived APIs

**Fig. 1:**
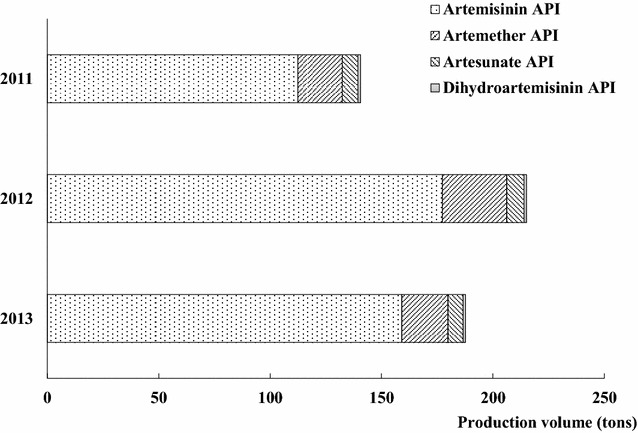
Production volume of artemisinin-derived APIs in China (2011–2013)

Between 2011 and 2013, the total production volume for artemisinin-derived APIs (artemisinin API, artemether API, artesunate API, and dihydroartemisinin API) reached 543.41 MT, among which artemisinin APIs accounted for the highest proportion (Fig. 1). The production volumes of artemisinin API accounted for 79.9, 82.4 and 84.8 % of the total production volume, while artemether API made up 11.0, 13.4 and 14.1 % of the total volume from 2011 to 2013, respectively. The proportion of both artesunate API and dihydroartemisinin API was less than 4 % during this period.

### Production of artemisinin-derived FPPs

**Fig. 2:**
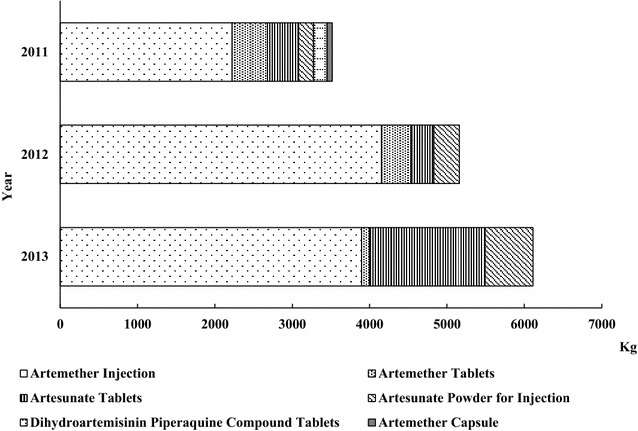
Production volume of artemisinin-derived FPPs in China (2011–2013)

During the same period, the total production volume of artemisinin-derived FPPs reported in China reached 14.79 MT, significantly lower than that of artemisinin-derived APIs. The production volume of artemisinin-derived FPPs continuously increased from 3515.63 kg in 2011 to 6112 kg in 2013 (Fig. 2).

Artemisinin-derived FPPs that China produced mainly included artemether injections, artemether tablets, artesunate tablets, artesunate powder for injection, dihydroartemisinin piperaquine compound tablets, and artemether capsules. Among those FPPs, artemisinin injection occupied the largest proportion of the total volume, accounting for 63.2, 80.5 and 63.8 %, respectively, during 2011 and 2013. Trends of production volume of different FPPs varied. The production volume of artesunate tablets and artesunate powder for injection showed a tendency of increase, while the artemether tablets decreased successively at the same period.

### Export of artemisinin-derived APIs

During 2012 and 2014, exports of artemisinin-derived APIs in China mainly included artemether API, artemisinin API, and artesunate API. The total export value and total export volume in three years reached US$ 207.12 million and 337.32 MT, however, both showed a tendency to decrease. The average unit price of artemisinin-derived APIs also showed a decreasing trend, with US$ 789.96/kg in 2012, US$ 537.27/kg in 2013, and US$ 429.08/kg in 2014. The export value almost halved in 2013 and 2014: 42.1 and 41.6 % of artemisinin-derived APIs exported to countries in Asia and North America, respectively, in terms of export values, followed by 13.2 % exported to Europe, while the proportions of exports to Africa and South America were only 2.9 and 0.2 % (Table 1).

**Table 1:** Exportation of artemisinin-derived APIs to various continents (2012–2014)

Year	2012		2013		2014	
	Export value (US$)	Export volume (Kg)	Export value	Export volume (Kg)	Export value	Export volume (Kg)
			(US$)		(US$)	
Asia	43,975,892	71,839	26,449,233	65,404	16,810,658	58,016
Europe	17,438,223	28,231	7844,286	17,445	1980,732	4571
Africa	4147,733	4930	1411,204	1925	544,503	2035
North America	45,562,096	35,671	21,980,184	22,595	18,648,316	24,142
South America	0	0	10,000	17	317,500	500
Total	111,123,944	140,671	57,694,907	107,386	38,301,709	89,264

Exports of artemisinin, artemether and artesunate are counted; the export volume and export value of dihydroartemisinin APIs was quite small and is not included

Among three types of artemisinin-derived APIs, artemether API occupied the largest proportion of the total export value, with US$ 110.94 million accounting for 53.6 % of the total amount during three years; while the export volume of artemisinin API ranked first in each year, though with a continuous decrease from 92.79 MT in 2012 to 52.75 MT in 2014. Artemisinin API ranked second in terms of export value and accounted for 44.4 % of the total amount in 3 years. It mainly exported to countries in Asia and Europe, accounted for 77.3 and 21.9 % of the total export volume of artemisinin API. The export volumes of artemether API reached 46.33 MT in 2012, decreased 29.6 % in 2013, followed by a 9.3 % increase. Artemether API mainly exported to countries in North America and Asia, accounted for 70.9 and 22.1 % of the total export volume of artemether API. The export volume and export areas of artesunate API in 3 years were quite limited, with only 4.90 MT and US$ 4.28 million-worth.

**Fig. 3:**
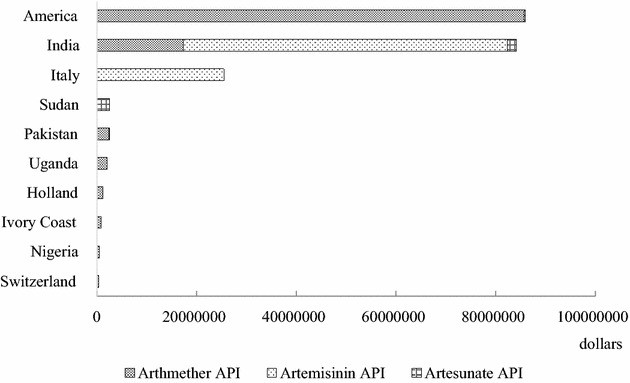
Export value of artemisinin-derived APIs to top ten countries (2012–2014)

The top ten countries in terms of the total export value during 2012 to 2014 were in Asia (India, Pakistan), North America (America), Africa (Sudan, Uganda, Ivory Coast, Nigeria), and Europe (Italy, Holland, Switzerland). The export value to America, India and Italy held the top three spots, accounting for 41.5, 40.6 and 12.3 % of the total amount in 3 years, respectively. The proportion of export value to other countries was all less than 1.2 %. The components of artemisinin-derived API exports varied among different countries. India and Italy mainly imported artemisinin API, while America mainly imported artemether API (Fig. 3).

### Export of artemisinin-derived FPPs

During 2012 and 2014, exports of artemisinin-derived FPPs in China mainly included artemether compound tablets, artesunate tablets, dihydroartemisinin tablets, and artemether injection. They were mainly exported to Africa, Europe, Asia, Oceania, North America, and South America. The total export value and total volume of artemisinin-derived FPPs in 3 years reached US$ 227.17 million and 4401.44 MT, more than the corresponding amount of artemisinin-derived APIs (US$ 211.39 million and 338.53 MT). In total, 73.46, 15.72 and 9.63 % of artemisinin-derived FPPs were exported to Africa, Europe and Asia in terms of the export value in 3 years (Table 2).

**Table 2:** Exportation of artemisinin-derived FPPs to various continents (2012–2014)

Year	2012		2013		2014	
	Export value (US$)	Export volume (Kg)	Export value	Export volume (Kg)	Export value	Export volume (Kg)
			(US$)		(US$)	
Africa	45,537,092	790,251	62,777,364	1336,523	58,577,658	1454,035
Europe	11,076,351	144,318	12,248,673	171,756	12,394,925	230,441
Asia	12,574,362	97,763	4504,684	62,511	4807,995	83,798
Oceania	834,354	12,199	834,321	8792	456,739	5851
North America	244,718	287	58,875	199	36,326	261
South America	16,130	57	162,278	2306	31,620	88
Total	70,283,007	1044,875	80,586,195	1582,087	76,305,263	1774,474

Artemisinin-derived FPPs include artemether compound tablets, artesunate tablets, dihydroartemisinin tablets, artemether injection

Compared to the decreasing trend of artemisinin-derived API exports, the export value of artemisinin-derived FPPs each year kept at a relatively stable level and the export volume increased with an annual growth rate of 51.41 % in 2013 and 12.16 % in 2014. In 2012, the total export value of APIs was 1.58 times that of FPPs in 2012, while the total export value of FPPs exceeded that of APIs in 2013 and almost doubled that of APIs in 2014.

**Fig. 4:**
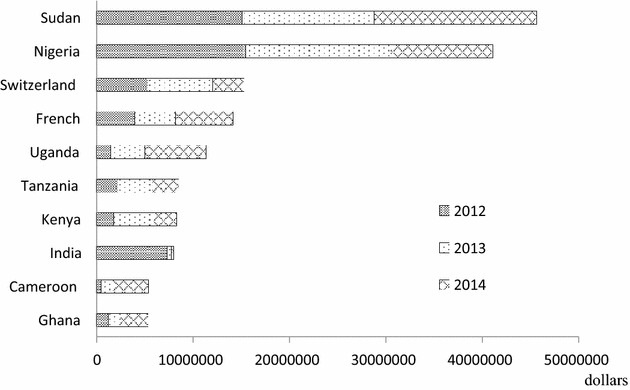
Export value of artemisinin-derived FPPs in various countries (2012–2014)

During 2012 and 2014, seven of the top ten countries with the largest export value of artemisinin-derived FPPs were concentrated in Africa, including Sudan, Nigeria, Uganda, Tanzania, Kenya, Cameroon, and Ghana. The export value of Sudan and Nigeria during 3 years reached US$ 45.59 million and US$ 41.03 million, together accounting for about 38.13 % of the total amount and exceeding that of other countries. Two European countries (Switzerland, France) and an Asian country (India) were also among top ten countries in terms of the total export value during this period, accounting for 6.7, 6.2 and 3.5 % of the total export volume in 3 years, respectively (Fig. 4).

Compared with export areas of artemisinin-derived APIs, export areas of FPPs were more widely distributed around the world and almost all countries with API imports also imported FPPs. Countries with both imports of artemisinin-derived APIs and FPPs produced by Chinese pharmaceutical companies were found in North America, South America, Africa, Europe, and Asia; countries only importing FPPs were concentrated in Africa, Asia and Oceania.

## Discussion

This study found that the total export value of artemisinin-derived APIs was close to that of artemisinin-derived FPPs during 2012 and 2014. However, the annually shrinking export value of APIs was in contrast to the gradually rising export value of FPPs. This indicated that exports of artemisinin derivatives is transforming from API-dominated export to more profitable FPP-dominated export.

China is continuously a major provider of artemisinin-derived APIs, occupying a share of 80 % in the global market [10]. Between 2012 and 2014, artemisinin-derived APIs produced by China were mainly exported to Asia and North America, where they were processed into FPPs and sold to malaria-endemic areas. America and India were the largest two purchasers of APIs, indirectly reflecting their ability in FPP processing from APIs, and their contribution to the FPP market. The recent rise of India in the global market of artemisinin-derived drugs should be attributed to the prequalification certification that various Indian pharmaceutical companies have obtained in the past few years and the low price of their generic drugs. Since Indian artemisinin-derived APIs first prequalified in 2008, significant progress has been made by Indian generic drug companies in prequalification certification [11]. The Global Fund to Fight AIDS, Tuberculosis and Malaria (GFATM) encourage grant recipients to purchase medicine from qualified manufacturers and suppliers based largely on price, leading to price competition and the advantage of Indian generic drugs over other original drugs [12].

This study showed that most of the artemisinin-derived FPPs produced in China between 2012 and 2014 were exported to Africa, the continent with the heaviest malaria burden. Apart from exports of artemisinin-derived FPPs passing WHO prequalification certification in the public market, other FPPs produced by China are retailed in the private market in Africa. It is easier for Chinese pharmaceutical companies to enter the African market through retailing, as pharmaceutical companies just have to pass local Goods Manufacturing Practice (GMP) certification and register drugs in local drug regulatory departments. However, the private market is quite small in comparison to the public market [13]. Certain European and Asian countries were also major importers of China’s FPPs, including Switzerland, France and India, corresponding to the headquarter locations of major pharmaceutical companies passing WHO prequalification certification (Novartis Pharmaceuticals, Switzerland, Sanofi, France, and some generics companies of India) [10]. Since malaria is not so prevalent in those countries, FPPs exported to these areas are usually transported or processed for further sale in other countries where malaria is endemic. The indirect sale of FPPs through qualified pharmaceutical companies in a third country poses limitations on profits made by Chinese pharmaceuticals. Moreover, a longer sales chain may raise the procurement cost and time for final consumers. From the perspective of global health, efforts are needed from China in the hope of benefiting more patients worldwide.

The decreasing export value of APIs and the increasing export value of FPPs seen in this research indicated a transformation from API-dominated export to more profitable FPP-dominated export. The decreasing price of artemisinin-derived APIs in the global market may facilitate the transformation of exportation structure, since it might act as a disincentive for Chinese producers of APIs and has a negative effect on the export volume. It may also be a contributor to the decreasing trend of the average unit price for artemisinin-derived APIs and further contributes to the decreasing export value of APIs. Besides, the new production of semi-synthetic artemisinin might also lead to the decrease of API exportation to Europe. Until recently, China is estimated to occupy a share of 3–5 % in the global FPP market [10]. WHO’s prequalification procedure is possibly the main obstacle for China to enter the global market of artemisinin-derived FPPs. The prequalification of medicine programmes launched by WHO in 2001 provided a recommended list of prequalified medicinal products for countries and organizations [14]. As the major purchasers of anti-malarial drugs, international organizations, such as WHO and GFATM, procure medicines from the list of prequalified medicinal products [15]. A small number of Chinese pharmaceutical manufacturers began to submit FPPs for WHO prequalification in 2004. Since then, only one Chinese pharmaceutical company has passed the prequalification certification for malaria FPPs.

In 2010, the prequalification of API was included in prequalification of the medicine programme. As a major contributor to global API production, China is well positioned to be a major source of prequalified APIs. In total, four Chinese artemisinin-derived APIs have prequalified so far [16]. The prequalification procedure for APIs sets the threshold for Chinese pharmaceutical companies to enter the API market and may be related to the shrinking export value of APIs from 2012 to 2014. On the other hand, it may be helpful for Chinese FPPs to pass the prequalification certification, since the prequalification process of FPPs can speed up using the prequalified APIs [17]. China’s potential to increase its presence in international markets of both artemisinin-derived APIs and FPPs is considerable and should be enhanced through greater participation in WHO prequalification.

Since India has made significant success in prequalification certification in recent years, a comparison with Indian pharmaceutical companies in this field can provide lessons and guidance for Chinese pharmaceutical companies in applying for prequalification certification. First, pharmaceutical companies in India are under strict supervision and regulation following the approaches adopted by the Food and Drug Administration, while most of Chinese pharmaceutical companies fail to meet the standards of the international GMP code [18]. Although the latest national GMP initiated in 2011 is in line with international GMP, China performs relatively poorly in actual quality regulation of pharmaceutical companies [19]. Therefore, corresponding incentive and disciplinary measures are needed to urge Chinese companies to instruct production strictly according to GMP standards. National companies should also deepen their understanding of WHO’s prequalification programme and actively apply for official evaluation and on-site inspection [20]. A prompt alternative to increasing market share and profits is to further expand the private market in more countries through bilateral trade and include Chinese medicine on the procurement list of various countries.

Although this study used currently the most accurate and official data, several limitations should be mentioned. First, the different sources of production and export data caused difficulties for comparison. Since the production data were self-reported by certain pharmaceutical companies, the data might be misstated, omitted or merged, and may not cover all enterprises. While the export data recorded by GAC included all the export goods and can reflect the overall exportation situation. Second, due to the availability of most recent data, the production data used in this study were from 1 year earlier than the export data. As there is time gap for produced drugs to be exported to foreign countries, the impact of time inconsistency will be reduced to a certain extent. Also, the total weight of FPPs might include the weight of other partner drugs or packaging. However, due to the stable standard requirements, the results should illustrate the actual tendency of changing for artemisinin. Since these data were the governmental official data available in China and used for policymaking, the results should provide enough information for further discussion.

## Conclusions

Between 2012 and 2014, the transformation from API-dominated export to more profitable FPP-dominated export showed an encouraging trend. In spite of challenges of prequalification procedure facing Chinese companies in the global market of artemisinin-derived medicine, the awarding of Nobel Prize to Tu Youyou for the discovery of artemisinin may stimulate China’s development in this field and attract attention to the initial research and development procedure, as well as final market expansion by accelerating prequalification certification. Moreover, the strong commitment of the government to a pro-export policy in this field and support from relevant agencies is of great benefit to Chinese manufacturers and will guarantee China’s participation in the global fight against malaria.

## Abbreviations

ACT: artemisinin-based combination therapy
APIs: active pharmaceutical ingredients
DALYs: disability adjusted life year
FPPs: finished pharmaceutical products
GAC: General Administration of Customs
GMP: Goods Manufacturing Practice
MIIT: Ministry of Industry and Information Technology
MT: metric tons
WHO: World Health Organization
